# Study of Tetrapodal ZnO-PDMS Composites: A Comparison of Fillers Shapes in Stiffness and Hydrophobicity Improvements

**DOI:** 10.1371/journal.pone.0106991

**Published:** 2014-09-10

**Authors:** Xin Jin, Mao Deng, Sören Kaps, Xinwei Zhu, Iris Hölken, Kristin Mess, Rainer Adelung, Yogendra Kumar Mishra

**Affiliations:** 1 Functional Nanomaterials, Institute for Materials Science, Christian-Albrechts University of Kiel, Kiel, Germany; 2 Synthesis and Real Structures, Institute for Materials Science, Christian-Albrechts University of Kiel, Kiel, Germany; Harbin Institute of Technology, China

## Abstract

ZnO particles of different size and structures were used as fillers to modify the silicone rubber, in order to reveal the effect of the filler shape in the polymer composites. Tetrapodal shaped microparticles, short microfibers/whiskers, and nanosized spherical particles from ZnO have been used as fillers to fabricate the different ZnO-Silicone composites. The detailed microstructures of the fillers as well as synthesized composites using scanning electron microscopy have been presented here. The tensile elastic modulus and water contact angle, which are important parameters for bio-mimetic applications, of fabricated composites with different fillers have been measured and compared. Among all three types of fillers, tetrapodal shaped ZnO microparticles showed the best performance in terms of increase in hydrophobicity of material cross-section as well as the stiffness of the composites. It has been demonstrated that the tetrapodal shaped microparticles gain their advantage due to the special shape, which avoids agglomeration problems as in the case for nanoparticles, and the difficulty of achieving truly random distribution for whisker fillers.

## Introduction

The fabrication of synthetic materials always tries to learn from nature and compete with it in terms of quality and quantity. [1], [2], [3], [4], [5] For example, enormous interests have been devoted to study the amazing ability of gecko’s toes and fabricate devices that mimic them. [6], [7], [8] Not only can they run fast on any surfaces, but can also clean their toes without the help of water. Against intuitive judgment, gecko’s toe is not sticky like epoxy adhesive tape, but made of rigid material with low surface energy. To exactly mimic the gecko structure, appropriate selection of material in terms of processing and properties is very important issue. Silicone rubber, such as cross-linked Poly(dimethylsiloxane) (PDMS), has been an important material in the field of micro-fabrication such as the bio-inspired adhesive surfaces, due to its excellent biocompatibility, low surface energy and very easy processing procedures. [9] In case of fibrous structure, there is always a compromise between stiffness and the aspect ratio of the structures to prevent the fibers from collapsing. Using a material of higher elastic modulus, one can achieve finer fibril structures with larger aspect ratios, which can better mimic the gecko’s toe. [10], [11] In order to increase the relative low elastic modulus of PDMS, reinforcement of the polymer with fillers would be desired. After filling, suitable characteristics such as biocompatibility and low surface energy should be maintained. Apart from higher modulus, superior hydrophobicity is also much desired for the improved self-cleaning ability. [12] To increase the elastic modulus and hydrophobicity of PDMS polymer, appropriate filler materials are required, especially in terms of size and shape. It is therefore very important to study the influence of the shape and size of the filler materials on the overall properties of a composite material. In this respect, ZnO material is a very suitable candidate due to its excellent biocompatible behavior and versatility of fabricating different nano- and microstructures. [13], [14], [15], [16] In this research, our main intention is to compare the stiffness and hydrophobicity of PDMS composites filled with ZnO fillers of different sizes and shapes, which gives an insight into the special effect of filler shape on composite material with elastomer matrix.

It is well known that the properties of polymers can be improved by the incorporation of rigid particles or fibers. The term “reinforcement” refers to the striking improvement in terms of stress-strain behavior, tear and abrasion resistance, which is induced by the presence of fillers in the polymer matrix, such as the addition of carbon black to natural rubber. In order to improve the properties of the composite, appropriate choice of filler material is necessary and parameters like size, shape, surface chemical structure of the fillers play very important roles. Various inorganic filler materials have been employed to reinforce polymer matrix, such as nano- and micro- SiO_2_, Al_2_O_3_, Mg(OH)_2_, CaCO_3_, glass fiber, carbon fiber, etc. [17], [18], [19], [20], [21], [22], [23] Traditionally most of the polymer composite materials can be classified by the shape of fillers as follows: particulate composites (fine particles), fiber composite (continuous or discontinuous fiber; long or short fiber or even shorter whiskers) and structural composite (laminates and sandwich panels). [24], [25] For particulate composite, size of the filler has been found to be the most important factor and it is shown that below a certain critical size (e.g., 30 nm diameter), the Young's modulus of the composite increases dramatically with the smaller size. [26] The nanosized particulates benefit from their high surface to volume (S/V) ratio on the better filler-polymer interaction, however, they suffer from the general problem of agglomeration. [27] Homogeneous distribution of these nanoparticles is highly desirable but very difficult through the existing techniques and is also the main limiting factor for their application as reinforcement fillers. [28] In the case of long fibers as fillers, one drawback is that the reinforcing effect is not isotropic. It becomes more significant when the fibers are continuous and arranged in a preferred orientation. Despite that the long fiber can share more load than short fiber, they are normally not suitable in important fabrication method for polymeric product such as injection molding and extrusion molding. [28], [29] Instead of long fibres, short fiber reinforcement gains its advantage in terms of cost and easy fabrication but the achievement of truly random oriented filler distribution is still difficult and remains an open challenge to further improve the reinforcement.

**Figure 1:**
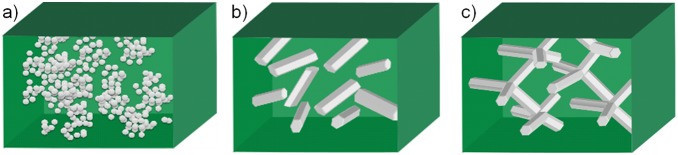
Illustration of the sample composites filled with different kinds of fillers. a) nanosized spherical ZnO particles (S-ZnO), b) microsized ground ZnO short fibers/whiskers (G-ZnO) and c) microsized tetrapodal ZnO particles (T-ZnO).

Recent developments in nanotechnology have added further dimensions for fabrication of different reinforced composites, due to new types of nano- and micro- scale fillers with excellent physical and chemical properties. [28], [29], [30], [31] Utilization of carbon nanotubes (CNT), as well as layered silicates as fillers have particularly gained enormous interests in the field of polymer composites reinforcement and have shown promising results. [32], [33], [34], [35], [36] Studies have revealed that the high aspect ratio of both kinds of fillers contributes to their extraordinary reinforcement for the composite. However, the issues like anisotropy, agglomeration and high cost of synthesis are still major problems and appropriate alternative fillers are desirable. ZnO material is probably one of the most investigated inorganic material in last decade because of its excellent multifunctional properties caused by, e.g., wide direct band gap (∼3.37 eV), large exciton binding energy (∼60 meV) and hexagonal wurtzite crystal structure which facilities easy growth of its one dimensional and several other types of nano- and micro- structures. [13] Use of ZnO structures as fillers in the composites exhibits double advantages. Firstly, the mechanical and other properties of the composites will be improved because of filler infiltration. [37] Secondly, the interesting physical and chemical properties of these ZnO structures equip/introduce multifunctional properties in these composites, e.g., antimicrobial ZnO-PLA films, [37] luminescent self-reporting composite materials. [38] In addition, the recently introduced flame transport synthesis (FTS) enables the cost effective synthesis of different ZnO nano- and microstructures with various morphologies, e.g., nanoseaurchins, nanorods, nanowires, tetrapods, multipods and several others. [39] The grown ZnO structures, e.g., 1D needles, tetrapods etc. by FTS approach have already shown promising potentials in the direction of antiviral, photo-catalysis, joining the un-joinable polymer, UV-detection and other applications. [40], [41], [42], [43], [44], [45] Here, tetrapodal shaped ZnO microparticles are selected as fillers because of their sub-micron size (easy to process) and characteristic tetrapodal geometry. The tetrapodal structure exhibits four arms (originating from one nucleation core) pointing along ∼105.9° with respect to each other which leads to a concave shape in space. Its high aspect ratio in 3 directions enables random whisker-like distribution of fillers when mixed into a matrix. Growth mechanism of ZnO tetrapodal shape structures has already been discussed in the literature and same holds for flame transport synthesis approach too. [46] Such type of special fillers has also drawn some interests recently in polymer composite research, such as in improvement for wear resistance, microwave absorption, tensile strength, etc. [47], [48], [49], [50] However, a comparative study on properties of reinforced composites infiltrated with different size and shapes of ZnO fillers which reveals the functionality of this special shape has not been reported to the best of our literature knowledge. In this work, composites of ZnO microcrystals filled in PDMS matrix (as illustrated in Figure 1) were fabricated and their mechanical and cross-section surface wetting properties were studied. The function of three different fillers, (i) nanosized spherical ZnO particles (S-ZnO), (ii) microsized ground ZnO short fibers/whiskers (G-ZnO) and (iii) microsized tetrapodal ZnO particles (T-ZnO) on the properties of composites have been compared in order to gain an insight into the effect of the shape of fillers.

## Results and Discussion

### (A) Structural morphologies of fillers and reinforced composites

**Figure 2:**
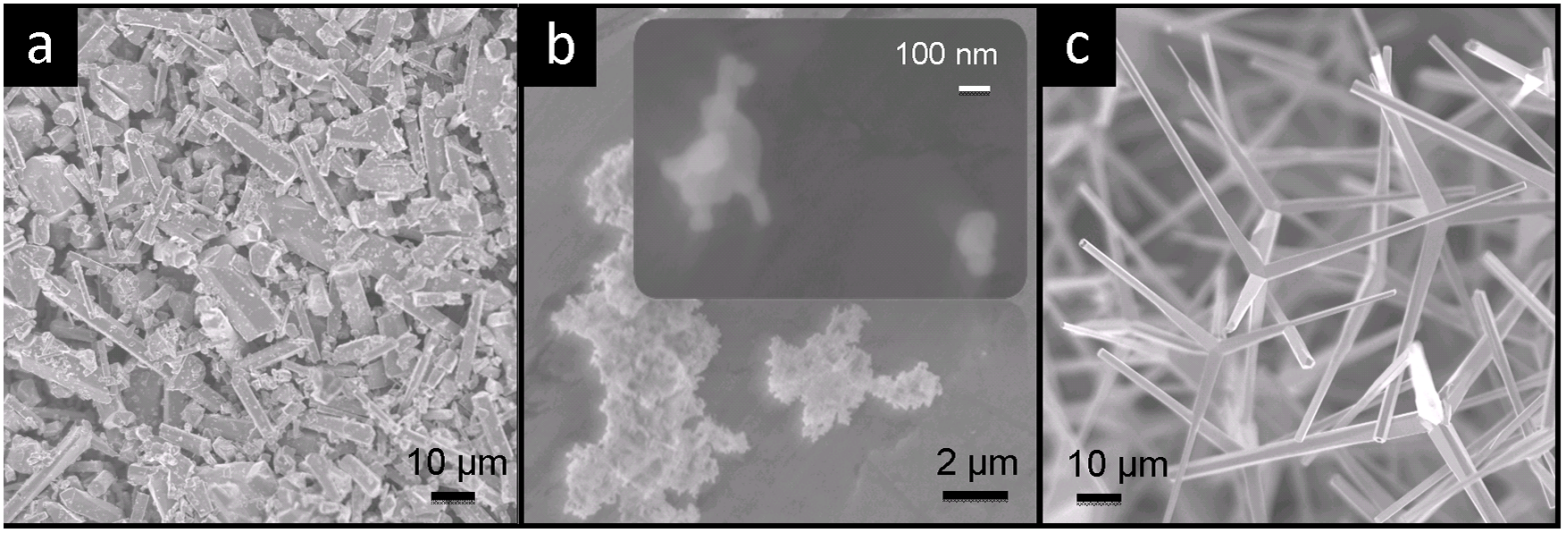
SEM images reveal the size and shape of different fillers used in the polymer composite. (a) ZnO microfibers and particulate, produced by grinding tetrapodal ZnO particles (G-ZnO). (b) Agglomerated ZnO nano spherical particles (S-ZnO), upper corner shows the magnified image. (c) Tetrapodal ZnO microparticles (T-ZnO).

Structural morphologies corresponding to G-ZnO, S-ZnO and T-ZnO fillers are shown by SEM images in Figure 2 (a–c). The G-ZnO type of filler consists of short fiber and particulates with aspect ratio approximately ranging from 1 to 10 (Figure 2a). The S-ZnO consists of spherical particles with diameter around 100 nm and due to their high surface area, they form microsized agglomerations (Figure 2b). A typical SEM image corresponding to T-ZnO filler is shown in Figure 2c. These tetrapods exhibit four arms pointing out from one core which leads to a concave shape in space. The angle between each two respective arms is mostly identical. The aspect ratio of each arm is ranging from 20 to 30. Due to the shape of T-ZnO, their agglomeration was prohibited and whiskers are well separated from each other, as can be clearly seen in Figure 2c. On the contrary, the G-ZnO particles can be packed more closely to each other.

**Figure 3:**
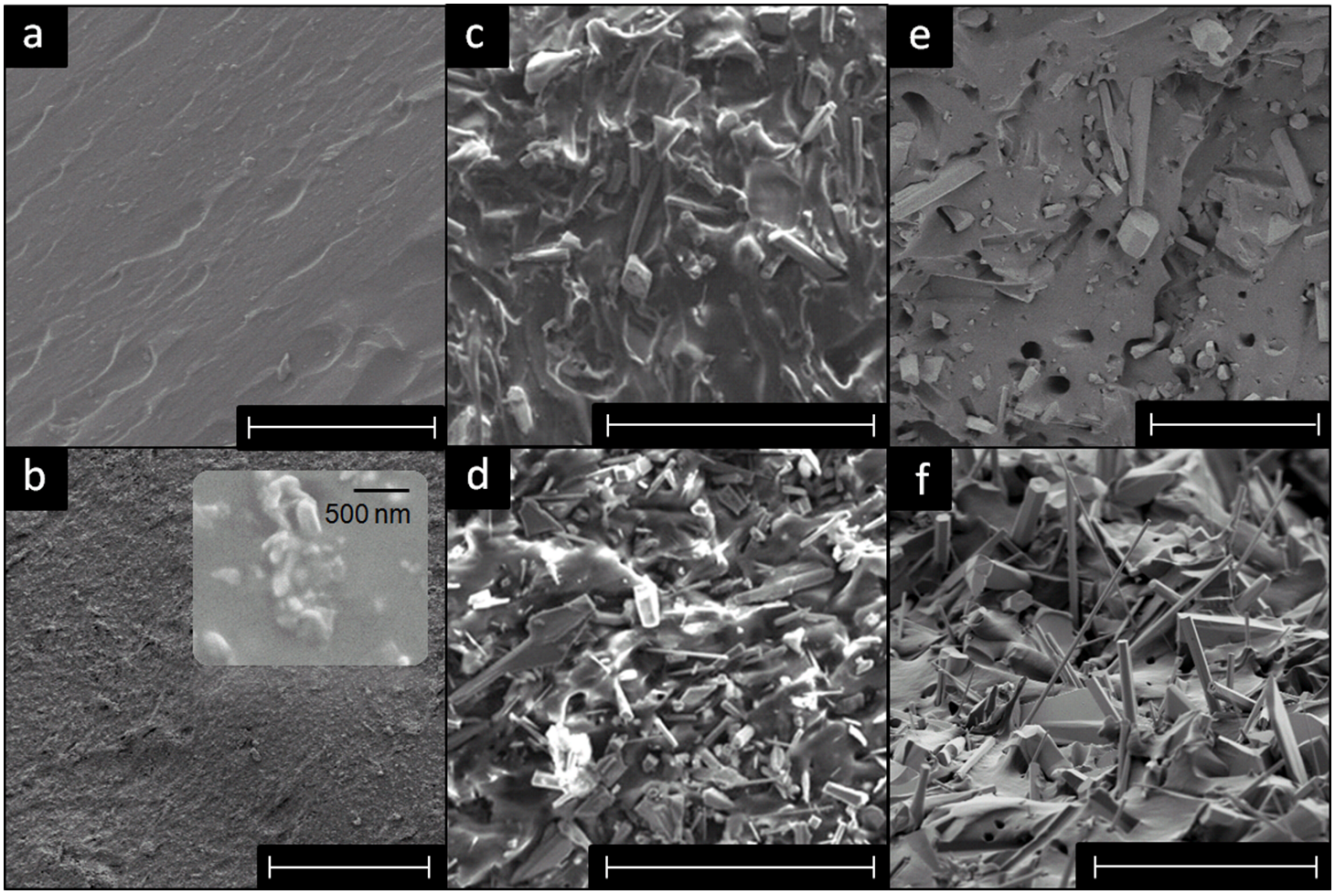
SEM images of the fabricated composites cross-sections. (a–d) are the cut cross-section and (e, f) are the torn cross-section (white scale bar indicates 50 µm): (a) the pure silicone (cross-linked PDMS) sample. (b) SEM image corresponding to Silicone filled with 50 wt% of S-ZnO and inset image in (b) is a high magnification view showing the nanoparticle agglomerates. (c) cut cross-section of silicone filled with 50 wt% of G-ZnO. (d) cut cross-section of silicone filled with 50 wt% of T-ZnO. (e) torn cross-section of silicone filled with 50 wt% of G-ZnO. (f) torn cross-section of silicone filled with 50 wt% of T-ZnO.

The fabricated composites (with different fillers) have been investigated in detail by SEM and cross-sectional SEM images of different composite samples are shown in Figure 3. It is observed from both cut cross-sections and torn cross-sections, that T-ZnO filled composites are relatively rougher in comparison to the specimen reinforced with G-ZnO (Figure 3c, d, e & f). For G-ZnO reinforced composites, it is found that whiskers are mostly laying along the surface. However, for T-ZnO reinforced composites, the arms of the tetrapods are pointing to different directions, which are especially pronounced in the torn cross-section (Figure 3f). In the cut cross-section, the T-ZnO are partially broken into short whiskers which is believed to be due to the cutting of the specimen during SEM sample preparation. For the S-ZnO filled composite, the nanoscale particles are hardly separable. As shown in magnified image in Figure 3c, the actual filler unit is rather the microsized agglomeration than the individual nanoparticles. Comparing to G-ZnO and T-ZnO filled composites, the cut cross- section of S-ZnO filled ones are relatively smoother on the micrometer scale level (Figure 3b).

### (B) Improvement in stiffness

**Figure 4:**
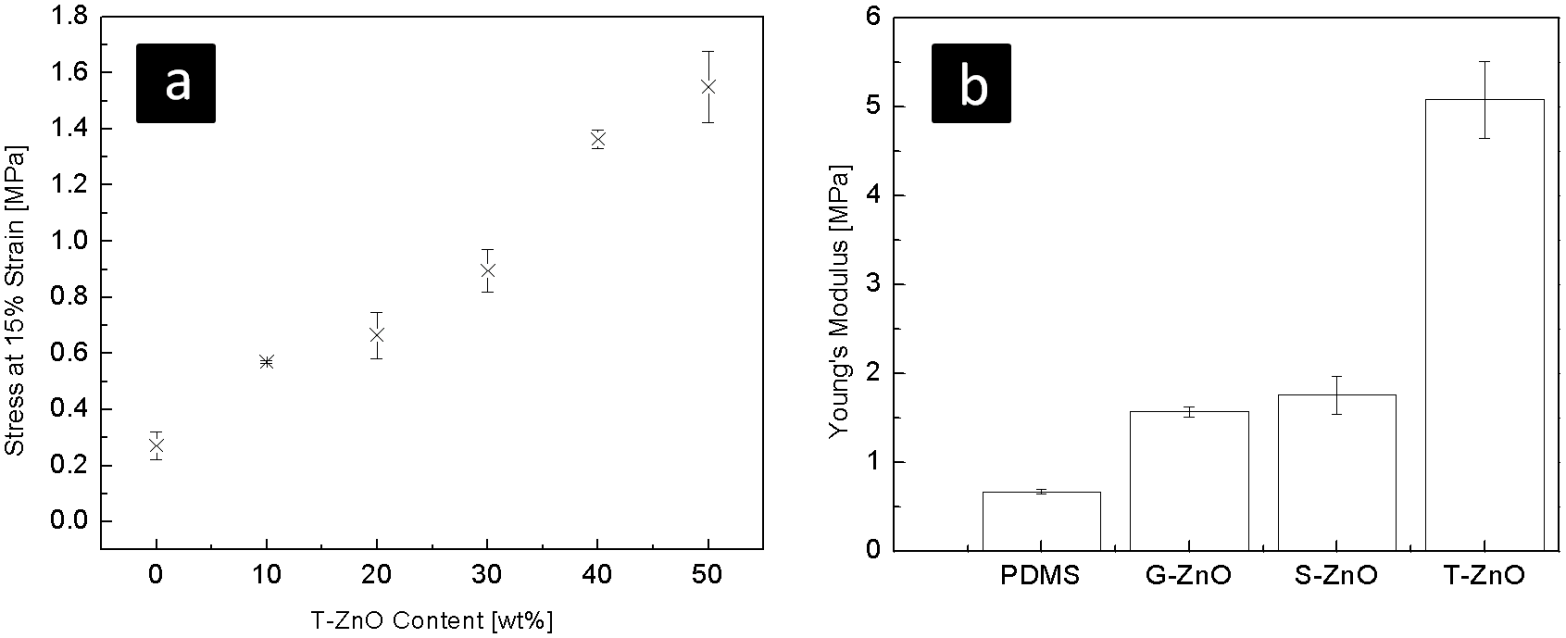
Tensile response of the fabricated composites. (a) Stiffness vs. filler content for T-ZnO. The stress at 15% stain is increased almost linearly as the T-ZnO content increase. (b) Stiffness vs. filler types. The Young’s modulus of control specimen (pure PDMS elastomer) and composites filled with 50 wt% of different fillers (G-ZnO, S-ZnO and T-ZnO) are plotted here. It is shown that all the fillers increase the Young's modulus of the PDMS, whereas T-ZnO gives the most distinctive result among all.

**Figure 5:**
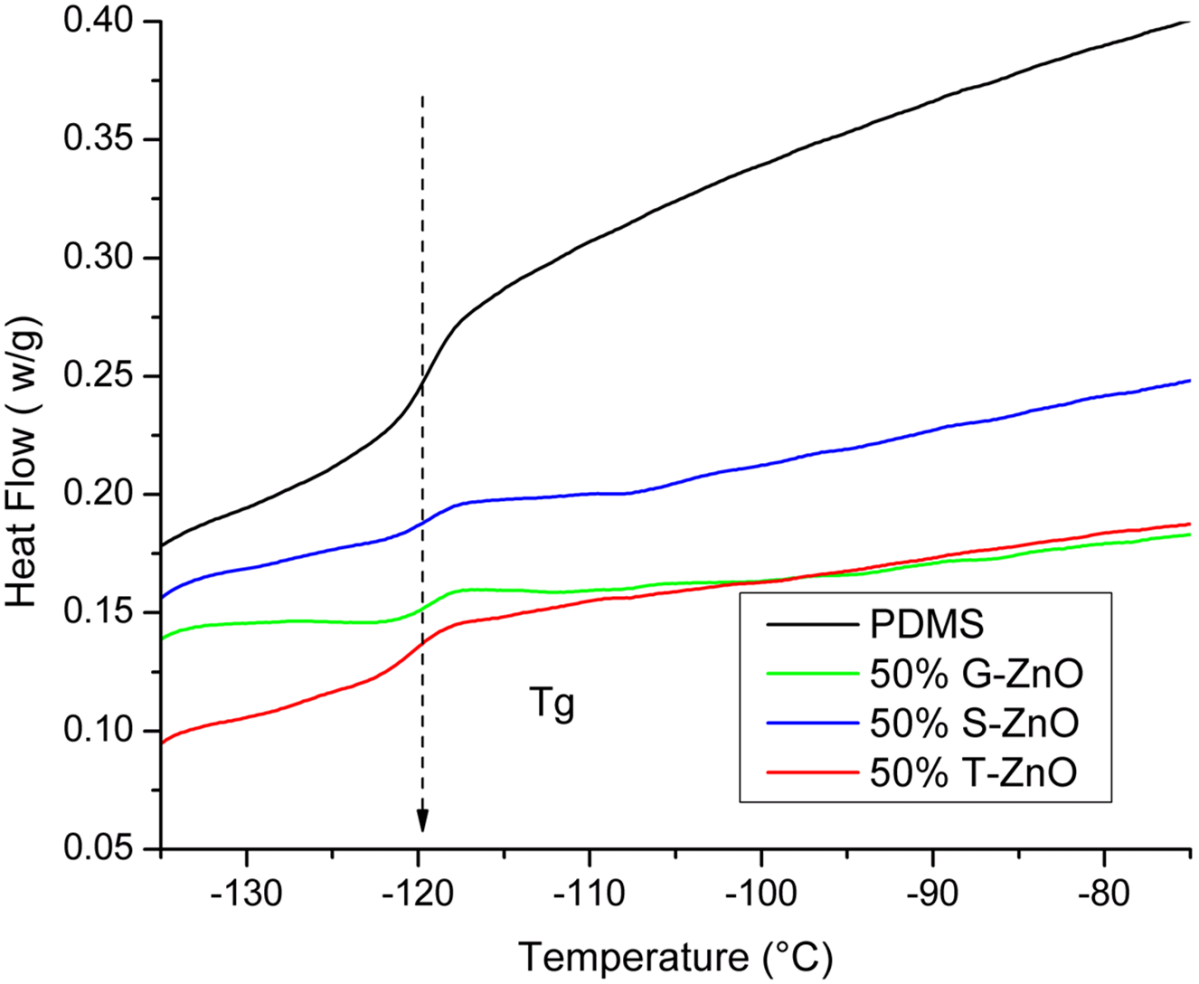
DSC measurement of T_g_. The heat flow is shown with baseline subtraction. Temperature scan rate is 10°C**/**minute. It can be observed for all three types of composite samples, the glass transition occur around the same value −120°C.

The tensile response of synthesized silicone composites reinforced with different ZnO fillers (up to 50 wt. %) has been measured and corresponding results are demonstrated in Figure 4. The stresses of composites with different filler content at the strain of ∼15%, are shown in Figure 4a and it shows that the stiffness of T-ZnO filled composite increases with the filler percentage. Figure 4b shows the filler shape dependence of the Young's modulus of the resultant composite and it has been observed that the T-ZnO reinforced composites exhibit the highest reinforcement. For each composition, three composite specimens have been examined and on the overall trend, the reinforcing effect increases proportionately with the filler content although the increase is not monotonic. This is most likely due to the homogeneity issue which is limited by the simple fabrication method. Elastomers exhibit glass transition temperatures (T_g_) far below room temperature. Therefore, the entangled polymer chains are able to be stretched and uncoiled from the lightly cross-linked network due to segmental rotation. The elastic modulus of polymer is increased by the fillers, simply due to the fact that the deformable volume is replaced by rigid parts. This also fits well with the experimental results of linear increase of stiffness with respect to filler fraction (Figure 4a) and no visible change in Tg value for three types of samples in comparison with the pure PDMS elastomer sample (∼120°C, Figure 5). It is known that the filler-matrix interface is a critical factor for the reinforcement and in our experiments no special surface coupling agent has been used to bind the filler with the polymer chains. The difference between the three fillers is mainly a physical effect induced by the shape. The T-ZnO has a higher aspect ratio than the other two types of fillers and the spherical nanoparticles (S-ZnO) have the highest surface to volume ratio. The ground ZnO particles (G-ZnO) have similar (slightly higher) surface to volume ratio than T-ZnO, but slightly less aspect ratio. Comparing the reinforcement in elastic modulus (Figure 4b), T-ZnO fillers show obviously the best performance.

The results shown in Figure 4 reveal that the filler having complex tetrapodal structure is very beneficial for the reinforcement in PDMS matrix. Beside the reinforcing mechanisms of whisker fillers introduced in many literatures, the tetrapodal fillers in present case require larger force to reorient and align to the direction of external force and thus further reducing the deformability of the composite material. Furthermore, due to the directional arms supported by the tetrapodal structure, T-ZnO are less likely to be densely packed with each other, or align themselves to the same direction, even at the high filling content (as shown in the torn surface in Figure 3f). In the case of S-ZnO, the high surface to volume ratio has the advantage of higher degree of filler-polymer interaction, but at the same time they exhibit the problem of agglomeration (Figure 2b & 3b) and hence the response for S-ZnO composite is slightly inferior to the case of G-ZnO filled composite.

### (C) Contact angle variation with T-ZnO filling fraction and different shape of fillers

**Figure 6:**
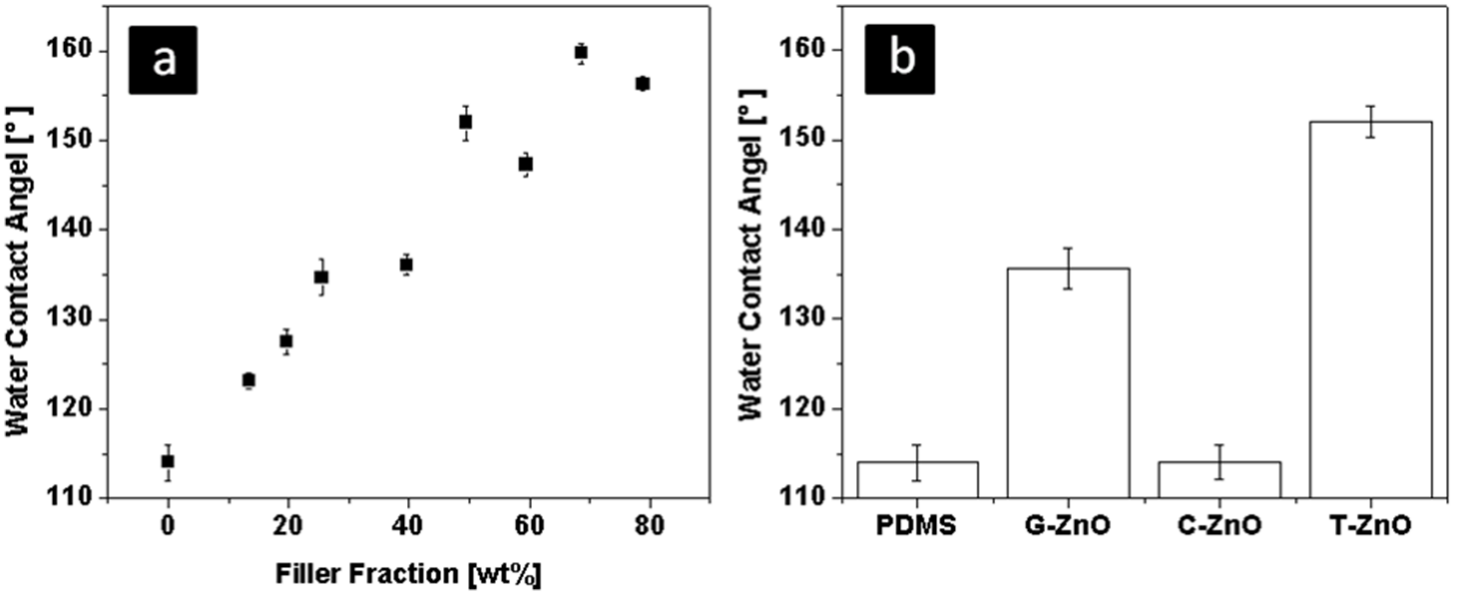
Wettability measurement results. (a) the water contact angle vs. filler content. It shows that the contact angle increases almost linearly with T-ZnO content. (b) the water contact angle verses different types of fillers at the same filling factor of 50 wt%. It is shown that the S-ZnO has no significant influence on the contact angle and T-ZnO gives the highest value.

The variation of water contact angle (WCA) on the sample cross-section with respect to the amount of T-ZnO mixed into PDMS is shown in Figure 6a. Without any filler, WCA for pure PDMS is 108°, which is hydrophobic. The contact angle of water droplet with the cross-section of the composites increases almost linearly with respect to the increase in T-ZnO filling fraction in the reinforced composite. The WCA of reinforced PDMS composites with different fillers (G-ZnO, S-ZnO and T-ZnO, each 50 wt %) is compared with that of pure PDMS sample and the results are demonstrated in Figure 6b. The contact angle of S-ZnO composite specimen is almost same as that of pure PDMS sample. In the case of composite sample filled with G-ZnO, an increase in WCA (from 114° to 136°) has been observed in comparison to S-ZnO sample. However composite sample reinforced with T-ZnO showed a significant improvement in wettability (WCA ∼148°) which is best among all the three types of fillers. Since the T-ZnO used in the experiments are hydrophilic, as shown by sink technique, the increased hydrophobicity cannot be only due to T-ZnO sitting on the surface. The variation in WCA can understood in terms of difference in roughness value in the composites filled with different fillers. Comparing the SEM images in Figure 3c and 3d, the T-ZnO samples exhibit the roughest cross-section. According to the Wenzel’s theory [51] of surface wetting, hydrophobic surfaces become more hydrophobic as the roughness increases. The S-ZnO particles induce roughness on nanoscale, which showed no apparent effect on the WCA.

### Conclusion

In summary, ZnO fillers of different size and shape have been used to modify the silicone rubber, in order to reveal the role of the shape of fillers on the properties of reinforced composites. Tetrapodal shaped microparticles, short microfibers and whiskers and nanosized spherical particles were successfully embedded in PDMS elastomer in form of composites in a straightforward method. The tensile elastic modulus and water contact angle of composites prepared with different types fillers have been measured. The results demonstrated that the tetrapodal shaped ZnO microparticles increase the stiffness and hydrophobicity of material cross-section on the highest level comparing to the other two kinds of fillers. Such properties of this polymer composite are highly desired in applications like biomimetics. It has been shown that the tetrapodal shaped microparticles gain the advantage due to their special shape. Such particular shaped fillers avoid the problem of agglomeration as in case of nanoparticles and the difficulty of achieving truly random distribution as in the case of short fiber fillers, therefore have shown the best results in both the reinforcement of tensile modulus and the reduction of cross-section surface wettability of the polymer composites.

## Materials and Methods

### (i) Materials and sample preparation

The silicone elastomer (PDMS, Sylgard 184) was purchased from Dow Corning Corporation. The ZnO tetrapods (T-ZnO) were synthesized by flame transport synthesis approach from Zn powder (diameter ∼5 µm from GoodFellow, UK) and polyvinyl butyral (PVB) powder (Mowital B 60 H from Kuraray GmbH, Europe) in an oven. [39] G-ZnO were obtained by grinding the T-ZnO in a ball milling machine at a rotation speed of 100 rpm. S-ZnO were purchased from Sigma Aldrich (CAS 1314-13-2, purity 99.99%). The composite samples were fabricated by mixing the fillers of different weight percentages into prepolymer mixture and curing afterwards. Initially, the two components of PDMS were mixed and the mixture was then degassed in a vacuum oven for about 5 min until no air bubbles observed in the solution. Then PDMS solution was dropped to the desired amount of ZnO particles (T-ZnO, G-ZnO, or S-ZnO). The mixture was gently stirred with a spatula till all the ZnO were mixed into the slurry. Afterwards the slurry was transferred to an Aluminum mold. Then it was degassed in the vacuum oven for about 15 min since it takes longer time for the bubbles to come out from a clay-like mixture than from the pure PDMS solution. Finally the mold is heated on a hotplate at 100*°*C for 40 min. After cooling down to ambient temperature, the cured samples were carefully removed from the mold. The reason for heating the mixture is that the long cure time (48 hours) at room temperature can cause precipitation of the ZnO, which might reduce their uniformity.

### (ii) Morphological investigation

The morphologies of different filler particles were investigated by scanning electron microscopy (SEM) instrument Zeiss Ultraplus (3 kV, 15 µA). The fabricated composites were also investigated inside SEM in detail to check their morphologies and distributions. The samples were prepared by *two* methods: either by cutting it through with a knife or tearing it apart after a crack is initiated by a cut. The revealed cross-sections were then sputter-coated with a few nanometer of gold to avoid the charging.

### (iii) Stress-strain response

Stress-strain measurements were carried out on a tensile testing machine (QuickTest QTS3). Most samples were extended at an elongation of 6 mm, which is c.a. 30% of the original length, in order to avoid error induced by sample slippage from the clamps. The strain rate was 60 mm/min. The dimension of the tested specimen region is 20×5×1 mm^3^. All measurements have been performed at room temperature. The tensile stress was calculated by the formula *σ = F/A_0_, where F* is the force applied on the specimen and *A_0_* is the cross section area.

### (iv) Differential scanning calorimetry (DSC) measurement

The measurement was carried out in a Differential Scanning Calormeter (Perkin Elmer Pyris 1), samples with c.a. 25 mg weight were used. Two temperature scan cycle per sample between −140°C to −70°C was carried out with rate 10°C/minutes. Results were subtracted with baseline measurement.

### (v) Wettability measurement

The wettability experiments were performed on a contact angle measurement machine (Dataphysics OCA5), where a camera can capture images of water droplets on different sample surfaces. The captured images were analyzed with image process software SCA20. The samples were cut into slices. A water droplet with a defined amount of volume was deposited on the cut surface of a slice. An image was captured immediately to avoid error induced by water evaporation. The wettability of T-ZnO was tested by sink technique. The T-ZnO were completely wetted by water and sunk in after being placed on top of water surface in a beaker.
